# Algal–bacterial bioremediation of cyanide-containing wastewater in a continuous stirred photobioreactor

**DOI:** 10.1007/s11274-024-04230-5

**Published:** 2025-01-09

**Authors:** Mona F. AbdelMageed, Marwa T. ElRakaiby

**Affiliations:** 1 The Biotechnology Center, Faculty of Pharmacy, Cairo University, Cairo, 11562 Egypt; 2 Department of Microbiology and Immunology, Faculty of Pharmacy, Cairo University, Cairo, 11562 Egypt

**Keywords:** Biodegradation, Phytotoxicity, Settleability, *Bacillus licheniformis*, *Chlorella* spp.

## Abstract

This study reports the isolation and characterization of highly resistant bacterial and microalgal strains from an Egyptian wastewater treatment station to cyanide-containing compounds. The bacterial strain was identified as *Bacillus licheniformis* by 16S rRNA gene sequencing. The isolate removed up to 1 g L^−1^ potassium cyanide, 3 g L^−1^ benzonitrile, and 1 g L^−1^ sodium salicylate when incubated as 10% v/v in MSM at 30 ℃. However, it failed to degrade potassium thiocyanate at all tested concentrations. The microalgal isolate was identified by electron microscopy as a strain of *Chlorella spp*.. Algal toxicity was tested by incubating the microalgae as 6% v/v in MSM containing 2 g L^− 1^ NaHCO_3_ with increasing concentrations of the pollutants. Results showed that 0.05 g L^−1^ KCN, 1.5 g L^−1^ benzonitrile, 5 g L ^−1^ KSCN, and 5 g L^−1^ sodium salicylate inhibited 93%, 96%, 75%, and 21% of algal growth, respectively. In a continuous stirred photobioreactor, the bacterial-microalgal microcosm detoxified synthetic wastewater containing 0.2 g L^−1^ KCN, 0.1 g L^−1^ benzonitrile, and 0.5 g L^−1^ sodium salicylate in 3.5 days of hydraulic retention time. System failure was recorded when the KCN concentration was increased to 0.25 g L^−1^. The effluent had no inhibitory effect on the germination of *Lepidium sativum* seeds in phytotoxicity testing. Temperature, pH, and chitosan effects were assessed on the algal/bacterial settleability. Statistical analysis showed no significant difference between the tested parameters. The microcosm represents a potential candidate for the treatment of industrial wastewater containing cyanide compounds.

## Introduction

Water scarcity is becoming a major life-threatening issue that needs urgent attention. Only 1% of total freshwater is available to overpopulated countries, especially in the Middle East (Abd-Elaty et al. 2022). The water deficiency problem may lead to political conflicts, as seen in many areas across the world (Bernauer and Böhmelt 2020).

Wastewater treatment (WWT) plays a critical role in the sustainable management of water resources by enabling the safe reuse of treated water while mitigating environmental contamination and preserving aquatic ecosystems. Industrial wastewater usually contains toxic compounds, while irrigation overflow may contain insecticides and other chemical fertilizers (Mainardis et al. 2022). Consequently, these wastewaters need special attention to alleviate their damage to the environment. Wastewater remediation strategies are diverse, where physical, chemical, and biological methods are the main broad approaches for WWT techniques (Rangel-González et al. 2024). The safest and most eco-friendly method is bioremediation (Azubuike et al. 2020). Its operation is clean and simple. Moreover, it diminishes the overall cost of pollutants’ detoxification, as biodegradation byproducts do not require further treatment. In addition, the resultant sludge is not as large in amount as in chemical or physical degradation methods and could be used in further beneficial applications (Mekuto et al. 2015).

Cyanide compounds are one of the most toxic pollutants in wastewater (Alvillo-Rivera et al. 2021). Cyanides are toxic to all forms of life, starting from microorganisms to humans and large animals (Alvillo-Rivera et al. 2021). Unfortunately, cyanides are discharged in a variety of industrial effluents, like plastics, gold mining, metal refining, pesticides, and pharmaceutical industries (Razanamahandry et al. 2017). In fact, cyanide is listed by the United States Environmental Protection Agency (US-EPA) as one of the priority pollutants (Environmental Protection Agency 1994). It is, therefore, pivotal to prevent their entry into the environment.

Biological treatment of cyanide-containing wastewater is an efficient alternative that avoids many of the drawbacks of chemical and physical methods such as high operational costs and secondary pollution risks (Aneyo et al. 2016). Biological approaches have shown promise due to their eco-friendly nature and cost-effectiveness. Among these approaches, fungi have shown significant potential in cyanide remediation due to their ability to degrade cyanide compounds. However, their application comes with certain limitations, such as slower degradation rates under specific environmental conditions and sensitivity to high cyanide concentrations (Kumar et al. 2017). Similarly, plants used in phytoremediation exhibit high tolerance and the ability to accumulate cyanide in their tissues. Nevertheless, this method faces challenges such as the slow rate of remediation, restricted scalability, and the need for careful species selection (Kumar et al. 2017).

Anaerobic bacteria can efficiently degrade cyanide; however, thiocyanate removal is challenging through anaerobic biological processes. Moreover, anaerobic treatment is generally slower and more prone to toxic disruptions caused by the presence of other compounds in the treated solution. Therefore, aerobic biological treatment processes are considered more effective for cyanide and thiocyanate remediation (Baxter and Cummings 2006).

The use of algal–bacterial symbiosis offers a more sustainable approach than solo bacterial detoxification for cyanide-containing wastewater. Studies proved that the integration of algal and bacterial systems achieved more efficient and advanced nutrient removal from wastewater compared to using either system independently. Additionally, this combined approach improved the recovery of biofertilizers during wastewater treatment (Fang et al. 2017; Tang et al. 2018). The algal–bacterial consortium operates through three primary mechanisms: artificial inoculation, utilization of natural substrates, and colonization (Saravanan et al. 2021).

Algae produce oxygen by photosynthesis, thus diminishing the need for mechanical aeration and reducing power supply costs. Simultaneously, bacteria assimilate pollutants, generating carbon dioxide (CO_2_) that is used in algal photosynthesis. In addition, nitrogenous byproducts are utilized by algae as nutrients (Johnson et al. 2020). Cyanide bioremediation using algal–bacterial microcosms can be achieved using the continuous stirred photobioreactor configuration. This setup ensures uniform mixing, enhanced mass transfer, and optimal light exposure, promoting efficient cyanide remediation (Altimari et al. 2022). Unlike batch or fixed-bed systems, the continuous stirred photobioreactor supports steady-state operation, enabling scalability and consistent performance. Unfortunately, extremely toxic pollutants, such as cyanide, can inhibit microbial growth. Formerly, the addition of cyanide to a photobioreactor for the treatment of sodium salicylate and phenol-containing influent led to system failure (Essam et al. 2014). Nevertheless, several organisms are capable of degrading cyanide compounds and even synthesizing them as a defense mechanism (Luque-Almagro et al. 2016).

Settleability refers to the ability of flocs (aggregates of suspended particles, including microorganisms) to settle under quiescent conditions within a defined period (Gerardi 2002). Poor settleability of biomass produced in biological reactors remains one of the most common operational problems such as increased costs for solids treatment, increased effluent solids concentrations, and increased risks to downstream ecosystems and public health (Schuler and Jang 2007). Factors influencing the formation and stability of the settleable algal–bacterial biomass include algal cell surface properties, extracellular polymeric substances (EPS) and the content of cations (Gutzeit et al. 2005).

This work reports the isolation and characterization of highly resistant bacterial and microalgal strains to selected cyanide-containing compounds from an Egyptian municipal wastewater treatment station. The biodegradation and/or tolerance capacities of the isolates were investigated. Eventually, the bioremediation of a mixture of the selected models of cyanides was optimized in a continuous stirred photobioreactor constructed by the symbiotic microcosm of the isolated bacteria and microalgae. The concentration of the pollutants in the influent was increased to reach the maximum degradation capacity of the photobioreactor (PBR). The concentrations of the pollutants in the effluent were measured by the respective analytical method of each chemical pollutant. The phytotoxicity bioassay was used to indicate the detoxification level of the treated effluent while settleability of the algal–bacterial flocs was evaluated to determine the efficiency of biomass separation and recovery.

## Materials and methods

### Isolation and characterization of a cyanide-degrading bacterial strain

#### Isolation of cyanide-degrading bacteria from a wastewater sample

The cyanide-degrading bacterial isolate was isolated from a wastewater sample collected from a WWT station in Egypt. The isolation was performed by the use of a selective enrichment technique with minor modifications (Potivichayanon and Kitleartpornpairoat 2010). A solution of 10% v/v of the sample in mineral salts medium (MSM) containing potassium cyanide (KCN) as the sole carbon source was prepared and incubated at 30 ± 2 ℃ in an incubator shaker (New Brunswick Scientific, Edison, NJ, USA) with shaking at 150 rpm for seven days. The sample was subcultured in KCN concentrations of 0.025 g L^−1^, 0.05 g L^−1^, and 0.1 g L^−1^. The isolation of the cyanide-degrading strain was accomplished by spreading it onto MSM medium containing 2% agar and 0.1 g L^−1^ KCN, then incubated at 30 ± 2 ℃ for seven days. After the incubation period, one isolated colony was picked and subcultured in MSM containing 0.2 g L^−1^ KCN. The isolated colonies were morphologically examined and further identified by conventional and molecular techniques.

#### Morphological examination of the isolate by transmission electron microscopy

The isolate was examined for morphological characteristics by transmission electron microscopy (TEM) at the Laboratories Complex, Faculty of Agriculture, Cairo University, Egypt. The sample was processed by negative staining technique and visualized by an electron microscope (Jem Joel, 1400, Japan).

#### Molecular identification of the bacterial isolate by 16S rRNA gene sequencing

The genomic DNA extraction and PCR amplification were performed using a Fast MicroSEQ 500 16S rDNA PCR Kit (Thermo Fisher Scientific, Waltham, USA). The primers were supplied with the kit and able to amplify the first 500 bp of the 16S rRNA gene. The PCR was performed using 30 cycles of initial melting step at 95 ℃ for 10 s, annealing at 64 ℃ for 15 s, final extension at 72 ℃ for 1 min, and final hold at 4 ℃. The PCR products were purified using ExoSAP-IT PCR Product Purification Reagent (Thermo Fisher Scientific, Waltham, USA) for sequencing. The sequencing was performed using Applied Biosystems 3500 Series Genetic Analyzer (Thermo Fisher Scientific, Waltham, USA) using the Sanger method by MicroSEQ 500 16S rRNA Sequencing Kit (Thermo Fisher Scientific, Waltham, USA). DNA extraction, DNA purification, PCR amplification, and sequencing were performed at Colors Lab, Cairo, Egypt. The generated sequence was then aligned using the basic local alignment search tool (BLAST) against the GenBank database on the NCBI website. A phylogenetic tree was created by MEGA X software using the Neighbor-Joining (NJ) method and the bootstrap values of the corresponding branches were analyzed.

#### Biodegradation capacity of the isolated strain for the selected pollutants

The biodegradation capacity of the isolated strain for cyanide pollutants was tested according to a method previously described with minor modifications (Mirizadeh et al. 2014). KCN, benzonitrile, potassium thiocyanate (KSCN), and sodium salicylate were added at 0.05 g L^−1^, 0.025 g L^−1^, 0.1 g L^−1^, and 0.5 g L^−1^, respectively, to a separate Erlenmeyer flask stoppered with a cotton plug and wrapped by parafilm to avoid hydrogen cyanide (HCN) gas loss by volatilization. The flasks contained 10% v/v of the isolated bacterial strain in MSM and were placed in an incubator at 30 ± 2 ℃ for seven days (Mirizadeh et al. 2014). Bacterial growth was observed by the presence of turbidity in the test suspensions as compared to the blank solutions. Samples were withdrawn every two days for analysis and compared to blank solutions free of the bacterial inoculum. The blank solutions were also tested for bacterial growth (turbidity) and pollutant concentrations (a decrease of 10% or less). The bacterial isolate was incubated with sequentially increasing concentrations of each pollutant to define the highest degradable concentration. The tested concentrations were as follows: KCN 0.1, 0.3, 0.5, 0.7, 1, 2 g L^−1^, sodium salicylate 0.5, 0.7, 1, 3 g L^−1^, benzonitrile 0.5, 1, 3, 4 g L^− 1^, and KSCN 0.05, 0.1 g L^−1^ (Table 1). The modified alkaline picric acid method was used to determine KCN concentration (Nwokoro and Dibua 2014), while the concentration of KSCN was determined spectrophotometrically with minor modifications (Degiampietro et al. 1987). The concentrations of benzonitrile and sodium salicylate were measured by HPLC analysis with minor modifications (Mradu et al. 2012).

**Table 1:** Biodegradation capacity of *Bacillus licheniformis* MMT1 when inoculated as 10% v/v in MSM and incubated at 30 ± 2 ℃ with different concentrations of the selected pollutants

Pollutant	Concentration (g L^−1^)/Duration of biodegradation (days)				
Potassium cyanide	0.1/5	0.3/7	0.5/9	0.7/9	1/9
Sodium salicylate	0.5/7	0.7/11	1/13		
Benzonitrile	0.5/7	1/7	3/17		
Potassium thiocyanate	0/11				

### Isolation and characterization of the microalgal strain

#### Isolation and identification of the microalgal strain

The microalgal strain was isolated from the same wastewater sample as that of the bacterial strain where 10 mL of wastewater were added to 100 mL of MSM containing 2 g L^−1^ sodium bicarbonate (NaHCO_3_), and incubated at 30 ℃ in an incubator shaker, with continuous illumination (5000–7000 lx) and agitation at 150 rpm for 72 h (Blaise and Férard 2005). LED lamps (30 W, Venus Electric LED lamps, Cairo, Egypt) were used for illumination. After algal growth was observed, 10 mL of the suspension were added to 100 mL MSM supplemented with 2 g L^−1^ NaHCO_3_, 0.5 g L^−1^ ampicillin, 0.08 g L^−1^ garamycin, and 0.064 g L^−1^ fluconazole then incubated under the same conditions for the isolation of a pure microalgal strain (Essam et al. 2013). The monoculture of the microalgal strain was examined by TEM (negative stain) and visualized by an electron microscope. The morphological characteristics of the microalgal strain were used for its identification.

#### Determination of algal toxicity by the selected pollutants

The microalgal strain was allowed to grow in the presence of increasing concentrations of each of the selected pollutants; then, algal toxicity testing was performed (Essam et al. 2013). The chlorophyll-a content of the microalgal strain was adjusted to 8–10 mg L^−1^ by chlorophyll-a content determination (Chen et al. 2011). Serial dilutions of the tested pollutants and the feed (Table 2) were prepared in MSM containing 2 g L^−1^ NaHCO_3_. The microalgal sample was added to the pollutants’ dilutions at 6% v/v (ElRakaiby et al. 2013). All the samples were continuously agitated at 150 rpm and illuminated at ~5000 lx by LED lamps for 72 h. Blank samples were prepared under the same conditions without adding the pollutants. Experiments were performed in triplicates. After test completion, 5 mL of each sample was withdrawn and analyzed for its chlorophyll-a content (Chen et al. 2011). The percentage inhibition of algal growth was calculated by comparing the chlorophyll-a content of the blank samples with the test samples according to the following equation:

$$\% inhibition = \frac{{Blank chl.a - Test chl.a}}{{Blank chl.a}} \times 100$$

$${\text{chl}}.{\text{a }} = {\text{ chlorophyll}} - {\text{a content}}$$

**Table 2:** Concentrations of the pollutants used in the algal toxicity testing with an algal inoculum of 6% v/v in MSM containing 2 g L^−1^ NaHCO_3_

Pollutant	Concentrations (g L^−1^)							
KCN	0.005	0.01	0.03	0.05	0.1	0.2	0.3	
Benzonitrile	0.1	0.5	1	1.2	1.5	2		
KSCN	0.1	0.5	1	1.5	2	3	4	5
Sodium Salicylate	0.1	0.5	1	1.5	2	3	4	5
Feed (Influent) (%)	6.25	12.5	25	50	100	Feed composition in g L^−1^: KCN (0.1), KSCN (0.06), Benzonitrile (0.1) and Sodium Salicylate (0.5)		

### Setting up the photobioreactor

The synthetic wastewater was prepared by mixing 0.1 g L^−1^ KCN, 0.06 g L^−1^ KSCN, 0.1 g L^−1^ benzonitrile, and 0.5 g L^− 1^ sodium salicylate in MSM. This mixture was referred to as *“Feed”* and supplied with 2 g L^−1^ NaHCO_3_ to allow for algal growth. The pH was kept around 7.5 to 8 using 2 N HCl. The PBR was first operated *“abiotically”* to exclude abiotic pollutants’ degradation (Essam et al. 2013). This phase was achieved by the addition of 0.5 g L^−1^ copper sulfate (CuSO_4_) to the synthetic wastewater for 14 days. The microcosm of bacteria and microalgae was formed by adding the bacterial strain and the isolated microalgae in a ratio of 1:3 v/v (Johnson et al. 2020). The feed container was connected to the system container by a commercial catheter. The established PBR was first operated for seven days without a change in the operating conditions to allow for acclimatization of the formed microcosm. The hydraulic retention time (HRT) was then gradually reduced to 3.5 days. The dark/light cycle was maintained at 12/12 h daily with illumination between 5000 and 7000 lx to simulate natural day/night conditions. The PBR was set up at room temperature and stirred at 150 rpm by a magnetic stirrer (Bibby, USA). Samples were regularly withdrawn from the PBR and the feed for analysis of the pollutants’ concentrations. Each time a parameter was changed, a period of 3–4 HRT was maintained. KCN concentration was increased gradually from 150 mg L^−1^ to 250 mg L^−1^ to evaluate the biodegradation capacity of the PBR.

### Algal settleability testing

The sedimentation ability of microalgae was evaluated using different parameters, including temperature (4 ℃ and 40 ℃), pH (3 and 9), and chitosan (0.5%, 1%, and 5%). The results were compared to the settleability of the PBR effluent (Essam et al. 2013). The standard testing conditions were adjusted to a temperature of 30 ℃ and a pH of 7. The test was conducted in 100 mL glass beakers filled with 50 mL of the algal-bacterial suspension and the tested flocculating agent or parameter. The tested suspensions were allowed to settle without any agitation or movement. A total of 5 mL samples were withdrawn at 1 cm below the surface of the suspension at 0, 10, and 30 min, then analyzed for chlorophyll-a content. Tests for each parameter were done in triplicate, and the results were taken as the mean value ± SD. Statistical analysis of the settleability results was performed by one-way ANOVA test to calculate significant differences between the means as compared with controls for each experiment at a 95% confidence level. Significance levels was set at p < 0.05.

### Phytotoxicity testing of the influent and effluent of the photobioreactor

The synthetic wastewater influent feed and treated effluent were tested for toxicity to plant seeds’ germination (Wang et al. 2001). Five seeds of *Lepidium sativum* were added onto a filter paper (5.5 cm, pre-sterilized) in a 6 cm diameter sterile Petri dish. A volume of 2 mL of the filtered sample, adjusted to pH 7, was added to the filter paper carefully, covered, and incubated at 30 ℃ for five days in darkness. Each test was performed in triplicate and compared to a negative control (tap water). Statistical analysis of the results was performed by one-way ANOVA test to calculate significant differences between the means as compared with controls for each experiment at a 95% confidence level. Significance levels was set at p < 0.05. Stem length was used in the determination of the toxic effect of the pollutants instead of the root due to the excessive branching of the root. The results were expressed as a percentage calculated as follows:

$${\text{Phytotoxicity }}\left( \% \right) = \frac{{A - B}}{A} \times 100$$

A = average stem length of control seeds (mm). B = average stem length of test seeds (mm).

## Results

### Identification of bacterial and microalgal strains

**Fig. 1:**
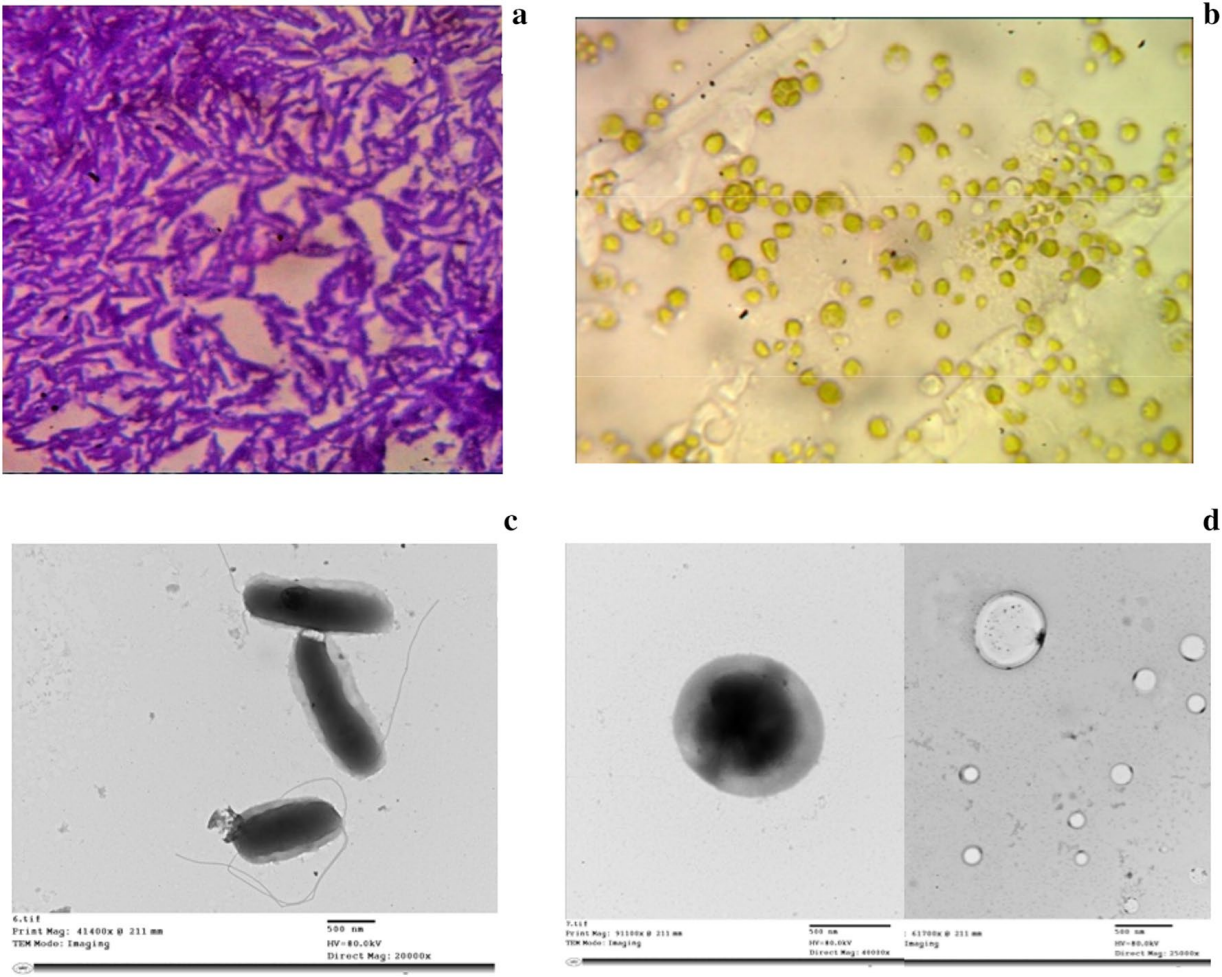
Microscopical examination (**a**) light microscopy of the bacterial isolate using Gram staining (Oil immersion lens, 100X), (**b**) light microscopy of a wet mount for the algal isolate (High power lens 40X); (**c**) transmission electron microscopy of the bacterial isolate and (**d**) transmission electron microscopy of the algal isolate via negative staining

**Fig. 2:**
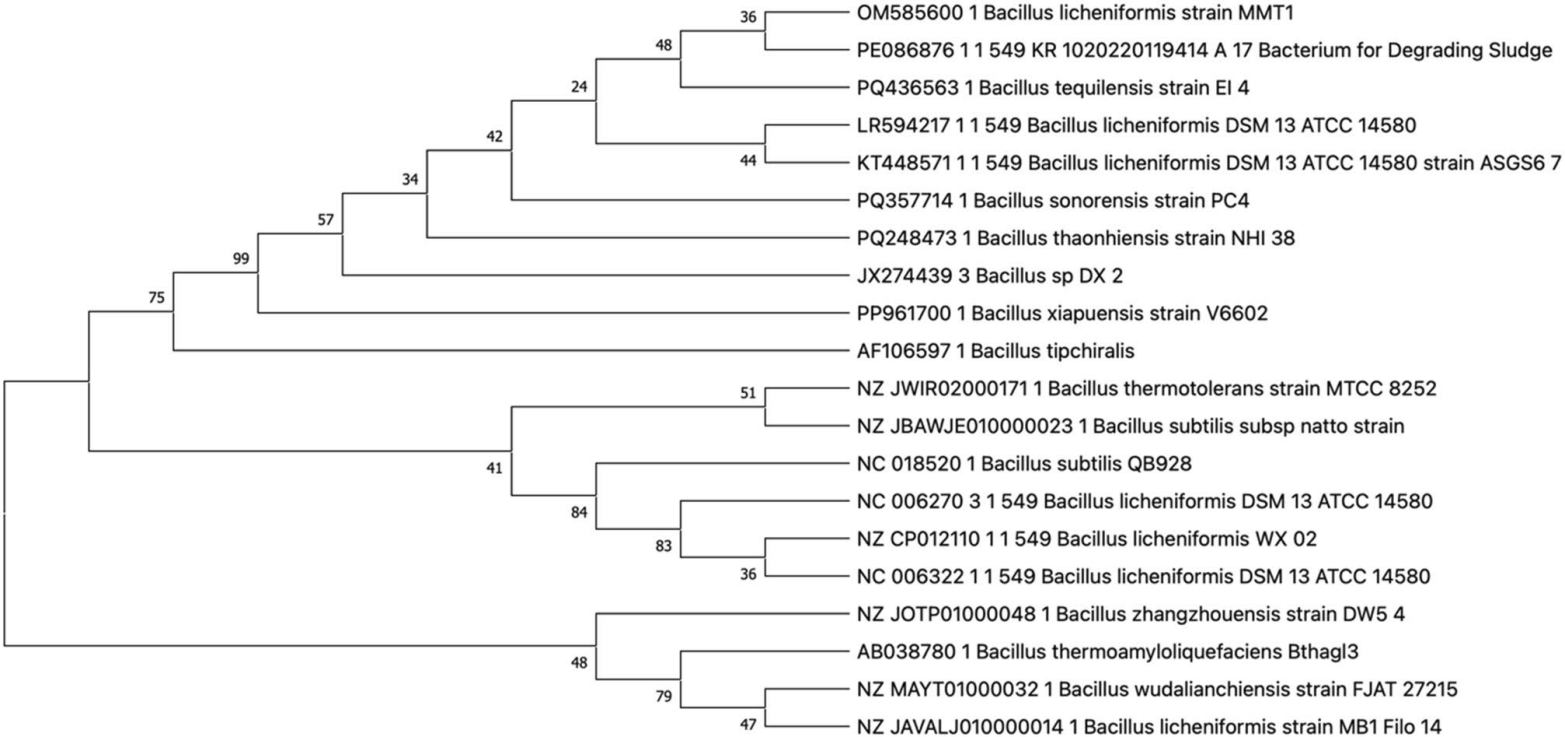
Phylogenetic tree of *Bacillus licheniformis* MMT1 and the strains of closely related species based on 16S rRNA sequences. Distances and clustering were performed using the neighbor-joining algorithm. The percentage of replicate trees in which the associated taxa clustered together in the bootstrap test (1000 replicates) are shown next to the branches. This analysis involved 20 nucleotide sequences. All ambiguous positions were removed for each sequence pair (pairwise deletion option). Evolutionary analyses were conducted in MEGA X.

Microscopical analysis of the isolated bacterial colonies after Gram staining showed motile Gram-positive rods with spore-forming ability (Fig. 1a). Further characterization using TEM with negative staining confirmed the presence of flagellated rods with a cylindrical outline (Fig. 1c). The molecular identification using 16S rRNA gene sequencing was performed. The phylogenetic tree showed that the 16S rRNA gene of the strain had a 100% bootstrap value to the 16S rRNA gene of *Bacillus licheniformis* strain MK12 (GenBank Accession No. JX068655.1). The sequence of the strain was subsequently deposited in the GenBank database with an accession number OM585600 and later named “*Bacillus licheniformis* MMT1” (Fig. 2). Microscopical examination of the microalgal isolate revealed identical small green spherical cells (Fig. 1b). When analyzed by TEM, the microalgal cells were globular with an un-sculptured wall; hence, the strain was classified to belong to *Chlorella* spp. (Fig. 1d).

### Algal toxicity testing

**Fig. 3:**
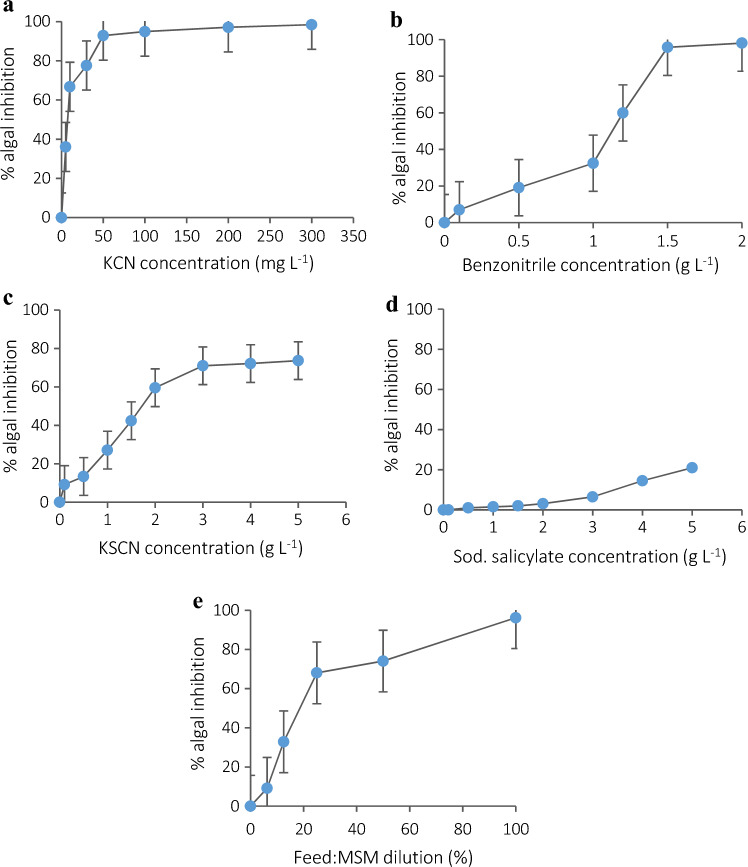
Algal toxicity testing as a percentage of algal inhibition using the selected pollutants at increasing concentrations of (**a**) KCN, (**b**) benzonitrile, (**c**) KSCN, (**d**) sodium salicylate and (**e**) the synthetic wastewater feed diluted with MSM of the photobioreactor

The ability of the microalgal strain to tolerate increasing concentrations of the pollutants was tested. The most toxic pollutant was KCN, where 0.05 g L^−1^ of KCN inhibited 93% of algal growth (Fig. 3a), while benzonitrile inhibited 96% of algal growth at 1.5 g L^−1^ (Fig. 3b). KSCN inhibited about 75% of algal growth at 5 g L^−1^ (Fig. 3c). Whereas the least toxic pollutant was found to be sodium salicylate, where 21% of algal growth inhibition was observed at 5 g L^− 1^ (Fig. 3d). The feed solution inhibited 96% of algal growth. When diluting the feed by 50% and 75% with MSM, the algal growth inhibition dropped to 74% and 68%, respectively (Fig. 3e).

### Biodegradation capacity of *Bacillus licheniformis* MMT1

**Fig. 4:**
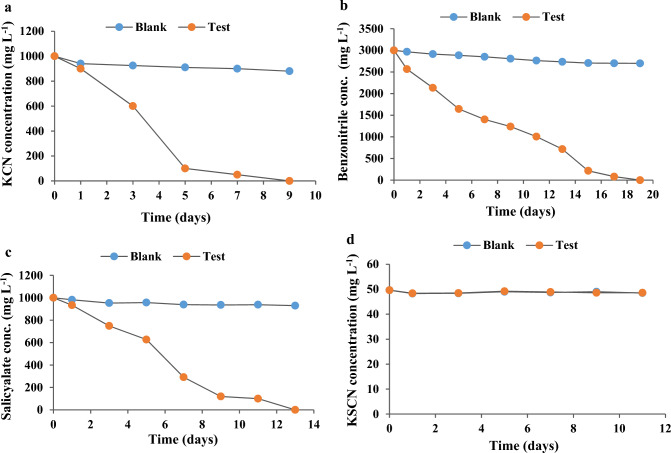
Biodegradation capacity (concentration/duration) by *Bacillus licheniformis* MMT1 of the selected pollutants (**a**) KCN, (**b**) benzonitrile, (**c**) sodium salicylate, and (**d**) KSCN

Preliminary testing revealed that *Bacillus licheniformis* MMT1 could degrade up to 50 mg L^−1^ KCN in 4 days, 25 mg L^−1^ benzonitrile and 500 mg L^−1^ sodium salicylate in 5 days. However, it could not biodegrade 100 mg L^−1^ of KSCN during the whole test duration. In order to find the maximum biodegradation capacity, *Bacillus licheniformis* MMT1 was incubated with increasing concentrations of each pollutant and was able to degrade up to 1 g L^−1^of KCN in 9 days (Fig. 4a) up to 3 g L^−1^ of benzonitrile in 17 days (Fig. 4b), and up to 1 g L^−1^ of sodium salicylate in 13 days (Fig. 4c). However, all the biodegradation tests of KSCN using 50 mg L^−1^ and 100 mg L^−1^ resulted in no change in concentration (Fig. 4d), hence, it was concluded that *Bacillus licheniformis* MMT1 could not degrade KSCN.

### Establishment of the photobioreactor (PBR) using the algal–bacterial microcosm

**Fig. 5:**
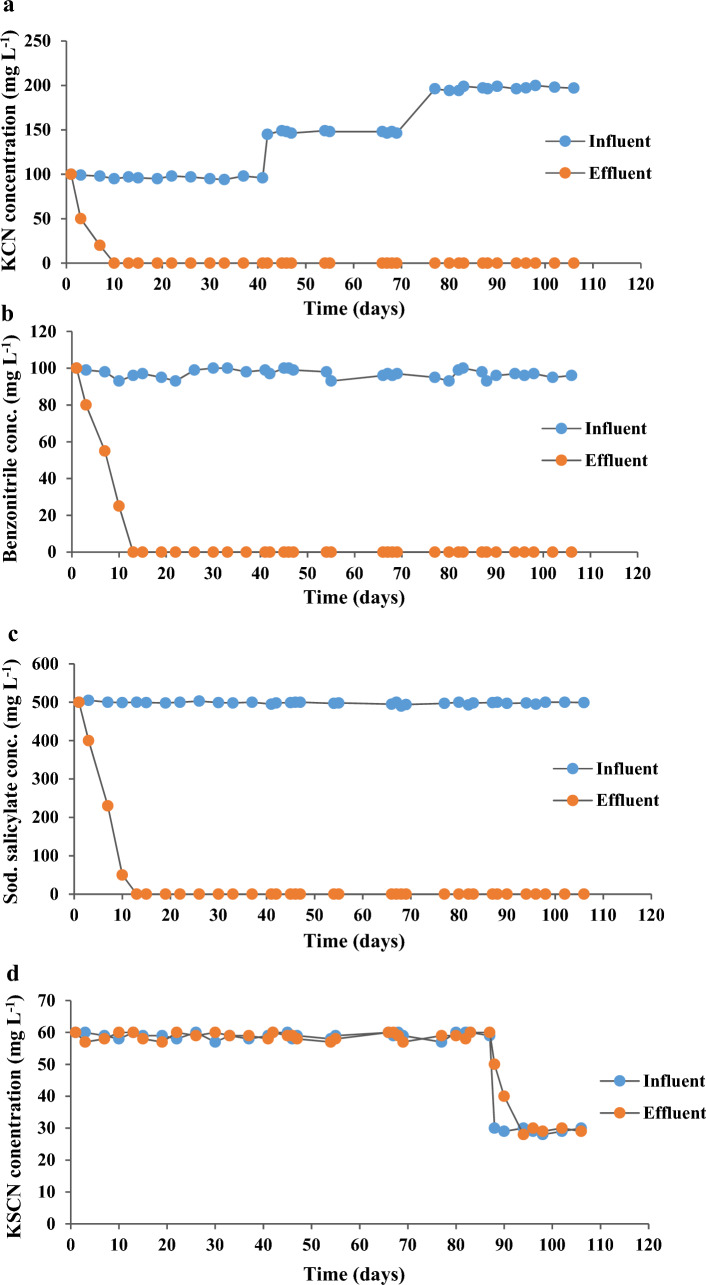
Pollutants removal (concentration/duration) in the photobioreactor over the 4 phases of operation (**a**) KCN, (**b**) benzonitrile, (**c**) sodium salicylate, and (**d**) KSCN

During the abiotic phase, the recorded change in the pollutants’ concentrations in the synthetic wastewater was less than 10% of their original concentrations, thus the absence of abiotic degradation was confirmed. The algal–bacterial microcosm was then introduced into the photobioreactor, and various parameters such as temperature, pH, and pollutants’ concentrations were recorded over 5 phases (Table 3 and Fig. 5).

**Table 3:** Parameters recorded in the PBR during its continuous operation for 106 days over five phases

	Day	T (℃)	pH	Agitation (rpm)	HRT (days)	KCN (mg L^−1^)	Benzonitrile (mg L^−1^)	Na Salicylate (mg L^−1^)	KSCN (mg L^−1^)
Initial feed	0	32	7.3	150	7	100	100	500	60
Phase I Feed	1	33	7.5	150	7	50	80	400	57
	3	30	7.4	150	7	20	55	230	58
	7	31	7.7	150	7	0	25	50	60
	10	33	7.8	150	6	0	0	0	60
	13	32	7.6	150	6	0	0	0	58
	15	30	7.5	150	5	0	0	0	57
	19	29	7.9	150	5	0	0	0	60
	22	29	7.8	150	3.5	0	0	0	59
	26	30	7.7	150	3.5	0	0	0	60
	30	31	7.7	150	3.5	0	0	0	59
	33	28	7.9	150	3.5	0	0	0	59
	37	28	7.5	150	3.5	0	0	0	58
	41	30	7.5	150	3.5	0	0	0	60
Phase II KCN (150 mgL^−1^)	46	30	7.8	150	3.5	0	0	0	59
	50	31	7.8	150	3.5	0	0	0	58
	53	30	7.9	150	3.5	0	0	0	57
	58	29	7.6	150	3.5	0	0	0	58
	62	29	7.6	150	3.5	0	0	0	60
	66	30	7.5	150	3.5	0	0	0	60
Phase III KCN (200 mgL^−1^)	70	32	7.9	150	3.5	0	0	0	57
	73	29	7.7	150	3.5	0	0	0	59
	76	30	7.7	150	3.5	0	0	0	59
	79	29	7.8	150	3.5	0	0	0	58
	83	29	7.8	150	3.5	0	0	0	60
	86	28	7.7	150	3.5	0	0	0	60
Phase IV KSCN (30mgL^−1^)	89	29	7.6	150	3.5	0	0	0	28
	91	30	7.9	150	3.5	0	0	0	28
	94	28	7.6	150	3.5	0	0	0	30
	98	30	7.6	150	3.5	0	0	0	29
	102	29	7.8	150	3.5	0	0	0	30
	106	30	7.9	150	3.5	0	0	0	29
Phase V KCN (250 mgL^−1^)	System Failure								

The color intensity decreases as the value of the cell decreases

#### Phase I: Acclimatization and initial operation

The microcosm was allowed to acclimatize to the influent feed for seven days. This period was then shortened to 3.5 days. The initial feed composition was: 0.1 g L^−1^ KCN, 0.1 g L^−1^ benzonitrile, 0.5 g L^−1^ sodium salicylate, and 0.05 g L^− 1^ KSCN in MSM. The HRT of 3.5 days was maintained for a duration of 4 times the HRT before the first change in the influent feed. The pH was adjusted to 7.5~8 throughout the entire period. The pollutants’ concentrations were measured, and the complete removal of all the pollutants was recorded after seven days of operation except for KSCN, where no change in concentration was detected.

#### Phase II: KCN concentration increment to 150 mg L.^−1^

Complete removal of KCN, benzonitrile, and sodium salicylate was recorded, while KSCN load remained unchanged.

#### Phase III: KCN concentration increment to 200 mg L.^−1^

Successful biodegradation of all the influent pollutants, except for KSCN, was detected.

#### Phase IV: KSCN concentration reduction to 30 mg L.^−1^

To allow for biodegradation of KSCN, its concentration was reduced from 60 mg L ^−1^ to 30 mg L ^−1^ in the influent feed. However, no change in concentration in the effluent was recorded. The analysis of the other three pollutants showed complete removal of their load from the effluent-treated water.

#### Phase V: KCN concentration increment to 250 mg L.^−1^

When the KCN concentration was again increased to 250 mg L ^−1^, algal growth was tremendously affected, and system failure was recorded. To restore the efficiency of the bioreactor, recycling of the treated effluent was performed; however, the biodegradation capacity of the reactor was not regained. A trial by fertilization of the reactor with increasing amounts of NaHCO_3_ from 4 g L^−1^ to 8 g L^−1^ was not enough to reboot the bioreactor to its ability to remove the pollutants, and eventually, the system washed out.

### Evaluation of the detoxification efficiency of the PBR by phytotoxicity testing

**Fig. 6:**
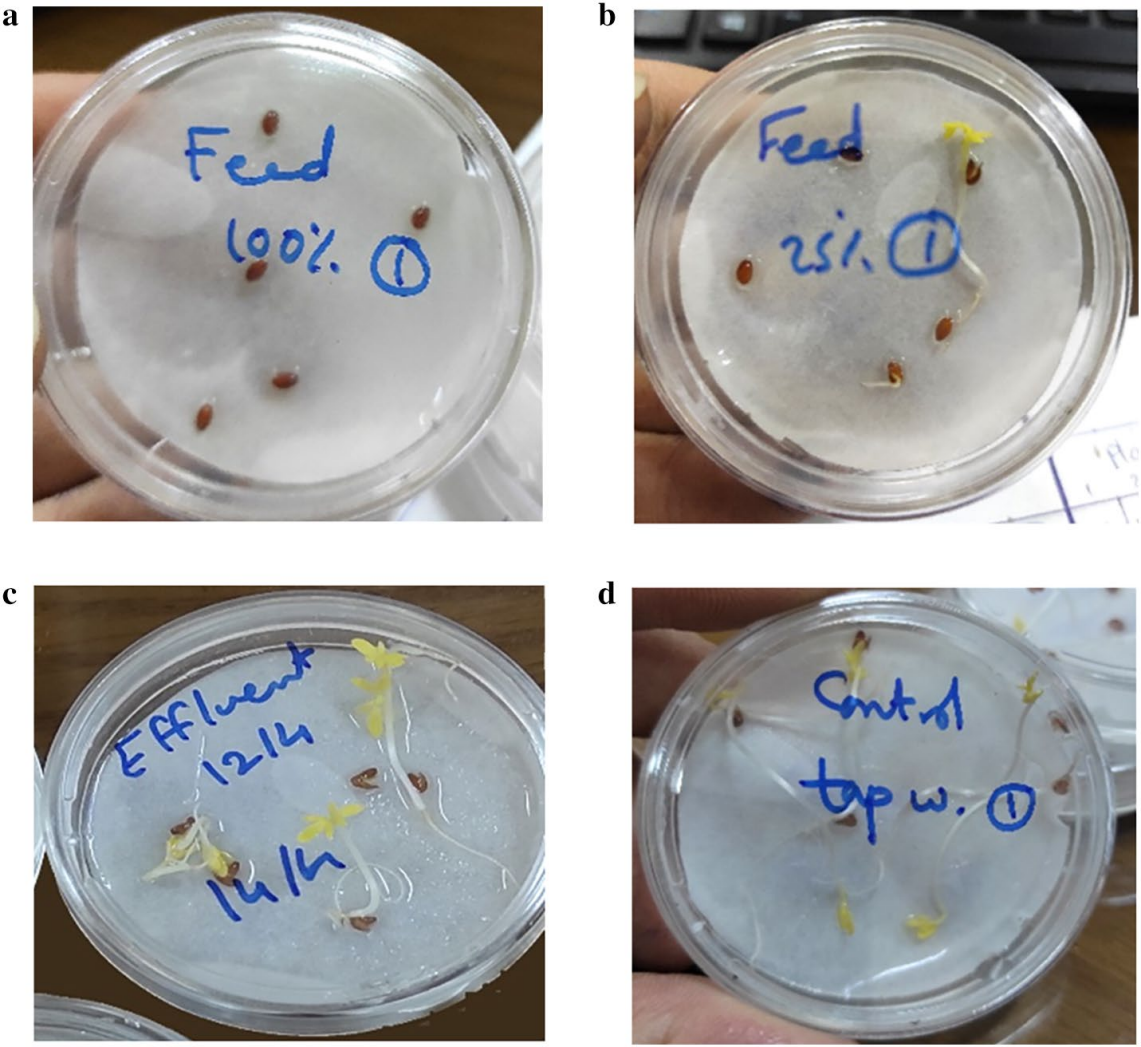
Phytotoxicity testing using *Lepidium sativum* seeds (**a**) total inhibition of the germination of the seeds in case of the undiluted influent feed, (**b**) inhibition of 88% of seed germination with a threefold dilution of the influent feed, (**c**) uninhibited germination of the seeds with the treated-effluent water, and (**d**) control plates with seeds germination using tap water

The synthetic wastewater inhibited 100% of the germination of *Lepidium sativum* seeds (Fig. 6a), while a threefold dilution of the influent feed inhibited the germination of the seeds by 88% (Fig. 6b). The effluent of the PBR, post-biological treatment, had no inhibitory effect on the germination of the seeds (Fig. 6c), where the length of the stems in the test plates was not significantly different from those recorded in the control plates (Fig. 6d).

### Algal settleability testing

**Fig. 7:**
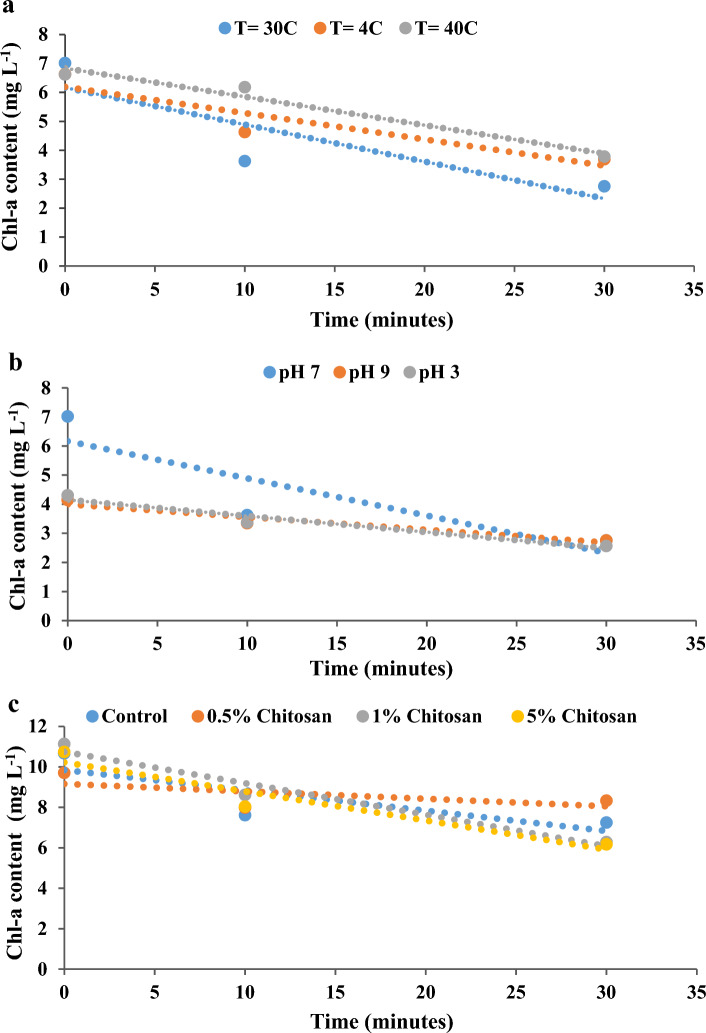
Settleability testing of the algal–bacterial flocs in the effluent-treated water under different conditions (**a**) temperature, (**b**) pH, and (**c**) chitosan

The flocculation ability of the algal-bacterial microcosm was tested alone (control) and under different conditions of temperature (4 and 40 ℃), pH (3 and 9), and chitosan (0.5, 1, and 5%) as a flocculating agent. Statistical analysis of the settleability tests showed that there was no significant difference between the tested parameters: temperature, pH, and chitosan and the naturally formed flocs (control) with p-values of 0.49, 0.38, and 0.88 respectively (Fig. 7).

## Discussion

Environmental pollution is a major concern worldwide, especially water pollution, affecting all forms of life (Boretti and Rosa 2019). Industrial and agricultural wastewater are the most abundant and hazardous sources of pollution. Therefore, WWT is a crucial and pivotal step before water discharge into the environment (Abd-Elaty et al. 2022). Cyanide compounds are toxic contaminants in heterogeneous types of industries, such as precious metals mining, coke-ovens, metal refining, pharmaceuticals, foods, biocides, and chemical and plastics production (Luque-Almagro et al. 2016). Sodium salicylate is a co-contaminant associated with some of the industries producing cyanide-contaminated discharges, such as pharmaceuticals, coke-ovens, and chemicals production. Sodium salicylate exerts an added toxicity to these discharges (Borisov et al. 2022). In the present work, KCN was used to evaluate the cyanide-degradation ability of the isolated bacterial and algal species in the presence of sodium salicylate as a co-contaminant. We successfully isolated a bacterial strain from a WWT station, *Bacillus licheniformis* MMT1, a Gram-positive motile rod with 99.22% similarity to *Bacillus licheniformis*.

Several studies reported the isolation of cyanide-degrading bacterial strains from various environments. For instance, Kandasamy et al. (2015) reported the coexistence of *Bacillus pumilus*, *Pseudomonas putida*, and *Bacillus cereus C2*, which were enriched in sodium cyanide and glucose-containing liquid media and isolated from a cassava factory wastewater (Kandasamy et al. 2015). Jiang et al. (2022) isolated *Aerococcus viridans* T1 from electroplating sludge, showing cyanide resistance up to 550 mg L^−1^ (Jiang et al. 2022). Similarly, *Pseudomonas* spp. such as *Pseudomonas putida* and *Pseudomonas stutzeri* were collected from coke-oven effluent, rich in phenol and cyanide (Singh et al. 2018). Additionally, *Pseudomonas parafulva*, isolated from polluted gold mine soil, was effectively applied for cyanide degradation (Moradkhani et al. 2018). To enhance degradation efficiency and spectrum, mixed microbial cultures have been employed. For example, Mekuto et al. (2016a) used a mixture of *Exiguobacterium acetylicum* and *Bacillus marisflavi* from the Diep River, Cape Town, to achieve co-degradation of cyanide and thiocyanate (Mekuto et al. 2016a). Similarly, mixed cultures of *Pseudomonas stutzeri* and *Bacillus subtilis* were employed for optimized cyanide compound degradation (Nwokoro and Dibua 2014).

Microalgae are diverse in terms of sizes, textures, colors, and shapes; therefore, their morphological characteristics can be used for taxonomical identification (Iamsiri et al. 2019). In fact, microscopic identification of microalgae is one of the most widely accepted and commonly used methods for microalgal species identification (Chong et al. 2023). Examination using light microscopy of the microalgal isolate revealed that it belonged to *Chlorella* species. This identification was corroborated by the TEM examination.

Microalgae has been used in WWT processes as a photosynthetic aeration source (Essam et al. 2014). Prominent algal species in WWT include *Chlorella* spp., *Scenedesmus* spp., *Oscillatoria* spp., and *Selenastrum* spp.. Particularly, *Chlorella* species are commonly used in WWT and detoxification processes (Coronado-Reyes et al. 2020). They are also applied in the biodegradation of pollutants symbiotically with biodegrading bacteria in photobioreactors (Mujtaba et al. 2018). In the present study, the *Chlorella* spp. strain was introduced in the PBR to provide oxygenation via photosynthesis, enabling aerobic degradation of the selected pollutants.

Previously, a microcosm combining *Chlorella vulgaris* MMI and *Pseudomonas* MTI efficiently treated simulated wastewater containing phenol and pyridine (Essam et al. 2013). In this system, *Pseudomonas* MTI was the primary agent responsible for the breakdown of phenol and pyridine, while *Chlorella vulgaris* contributed to oxygen production via photosynthesis, sustaining the bacterial degradation process (Essam et al. 2013). Similarly, Ryu et al. (2014) investigated thiocyanate removal using a consortium of bacteria (activated sludge) and microalgae in a sequential two-step process. Initially, bacteria facilitated the degradation of thiocyanate into nitrification products such as nitrite and nitrate. Subsequently, microalgae were introduced to assimilate and remove these byproducts, effectively complementing the bacterial degradation (Ryu et al. 2014).

Normally, microalgae are more sensitive to physical and chemical changes than other microorganisms (Omar 2010). The algal toxicity rank of the tested pollutants was as follows: KCN > Benzonitrile > KSCN > Salycilate. KCN, as a simple salt of hydrocyanic acid, releases free cyanide ions, which are highly toxic due to their ability to inhibit cytochrome c oxidase in the mitochondrial electron transport chain (Park et al. 2017). Benzonitrile, an aromatic nitrile, has reduced toxicity compared to KCN as its cyanide group is covalently bonded to a benzene ring, limiting free cyanide release (Sulistinah and Sunarko 2020). In contrast, the thiocyanate ion has lower toxicity than other cyanide compounds (Mekuto et al. 2016a). Sodium salicylate, an aromatic compound with a hydroxyl group, is non-cyanogenic, and its potential toxicity arises from other mechanisms like membrane destabilization or enzyme inhibition. Nevertheless, salicylates are considered one of the hazardous constituents present in industrial effluents (Borisov et al. 2022). These structural differences influence their behavior and toxicity in biological systems, which are reflected in their degradation profiles during the study.

In the present study, the algal toxicity of the influent feed containing a mixture of the selected pollutants was relatively higher than that of each single pollutant, where the feed inhibited ~ 96% of the algal growth. This may be attributed to the addition of a mixture of chemicals together having a similar mode of action to the feed. This mixture may produce an additive toxic effect that is larger than the effect of each component if applied individually (EC European Commission 2012). However, microalgae continued to grow during the operation of the algal–bacterial PBR, proving the efficient detoxification by the bacterial strain.

To test for the maximum biodegradation capacity, the bacterial strain was allowed to grow in increasing concentrations of each of the four pollutants using MSM as a culture medium with no carbon source. *Bacillus licheniformis* MMT1 was unable to degrade potassium thiocyanate but it effectively removed all other pollutants tested. The degradation of the higher concentrations of each pollutant showed a period of acclimatization in the degradation curve, where a duration of 12–24 h elapsed before recording a significant decline in concentration of each pollutant. This increase in degradation duration may be interpreted by the increase in the lag time needed by the bacteria to overcome the toxic effect of the pollutants and to activate the machinery of degrading enzymes in the presence of higher pollutant concentrations. Indeed, bacterial activity needs additional time for acclimatization with increasing pollutant loads (Mekuto et al. 2016b).

The adjustment of pH is an important step in cyanide treatment. To avoid volatilization of HCN gas from solution, alkali-tolerant microorganisms, or acclimatization of the degrading microorganism to the alkaline environment may be adopted (Vallenas-Arévalo et al. 2018). Airtight containers may also be used (Akinpelu et al. 2015). In this work, we used airtight Erlenmyer flasks with no shaking, and the pH was adjusted to 7.5–8 in order to minimize HCN loss, maintain the environment hospitable to algal growth in the PBR, and avoid salting out of the chemicals.

The capacity of bacterial degradation of cyanide-containing compounds varies from 0.15 g L^−1^ to ~ 0.5 g L^−1^ of cyanide or more. In one study, *Aerococcus viridans* degraded 84% of 0.2 mg L ^−1^ cyanide within three days and 87% of 0.15 mg L ^−1^ cyanide in 2.3 days. This degradation was accomplished by the addition of glycerol as a carbon source and peptone as a nitrogen source (Jiang et al. 2022). In another study, a *Bacillus licheniformis* strain degraded 98% of 0.5 g L^−1^ KCN in 5 days after its adaptation to an alkaline medium (Vallenas-Arévalo et al. 2018). In our work, *Bacillus licheniformis* MMT1 had the ability to biodegrade up to 1 g L^−1^ of KCN in nine days. This biodegradation capacity is among the highest reported for cyanide biotreatment (Table 4).

**Table 4:** Biodegradation capacity of previously reported strains vs *Bacillus licheniformis* MMT1 of the present study and their biodegradation conditions (temperature, pH, HRT, and isolation source)

Strain	Source	T (℃)/pH	Carbon/ Nitrogen source	Max capacity (g L^−1^)/HRT	References
*Aerococcus viridans*	Electroplating sludge	34 ℃/8	Both	0.2/3 days	Jiang et al. (2022)
*Pseudomonas parafulva* NBRC 16636(T)	Gold mine soil	32 ℃/9.9	Carbon	0.2/13 days	Moradkhani et al. (2018)
Community dominated by *Bacillus* spp.	Electroplating wastewater	30 ℃/9.9	–	0.25/2 days	Mekuto et al. (2018)
*Bacillus* spp.	Wastewater streams sediment	20 ℃/10.5	Carbon	0.3/10 h	Guadalima and Monteros (2018)12/17/2024 12:25:00 pm
*Bacillus licheniformis* adapted to alkaline pH	Abandoned gold mine	32 ℃/10	–	0.5/5 days	Vallenas-Arévalo et al. (2018)
*Bacillus licheniformis* MMT1	Municipal wastewater treatment station	32 ℃/7.5	–	1/9 days	The present study

The duration to completely biodegrade the influent load of 1 g L^−1^ KCN was around 9 days, which was relatively long compared to the time reported in previous studies of cyanide degradation. However, in our work, no pre-treatment steps nor additional external carbon or nitrogen sources were introduced in the degradation process. In addition, only a single cyanide-degrading strain, *Bacillus licheniformis* MMT1, was inoculated in the PBR, unlike other studies where a consortium of degrading bacteria was used to achieve complete removal of pollutants (Table 4).

Cyanide degradation can be accomplished via several possible enzymatic mechanisms: oxidative, reductive, hydrolytic, and substitution (Oshiki et al. 2019; Alvillo-Rivera et al. 2021). For *Bacillus licheniformis* MMT1 to effectively degrade the cyanide-containing pollutants (KCN and Benzonitrile), the strain may be producing enzymes such as cyanidase or nitrilase or both. These enzymes catalyze the breakdown of cyanide through hydrolysis. The substitution pathway, which involves the rhodanese enzyme, was ruled out as it requires the presence of thiosulfate in the medium. In addition, thiocyanate concentration did not increase during the operation of the PBR, indicating that no thiocyanate, needed for rhodanese activity, was newly formed. Therefore, it is unlikely that rhodanese contributed to the degradation observed in our work. *Bacillus licheniformis* MMT1 failed to biodegrade KSCN probably due to the absence of the specific enzymatic systems required for its degradation.

In contrast, the ability of *Bacillus licheniformis* MMT1to degrade sodium salicylate may involve the production of enzymes such as salicylate hydroxylase, salicylate 5-hydroxylase, or salicylate 1,2-dioxygenase, which are known to metabolize sodium salicylate through hydroxylation or cleavage into various metabolites like catechol, gentisic acid, or 2-oxo-3,5-heptadienedioic acid, respectively (Górny et al. 2019).

For the efficient bacterial removal of a mixture of contaminants, some techniques may be employed, such as the use of more than one biodegrading bacteria, the immobilization of the degrading bacteria, and/or the application of additional pretreatment techniques (e.g., adsorption) (Singh et al. 2016). For instance, to restore the biotreatment efficiency of an established PBR lost upon the addition of ~ 50 mg L ^−1^ cyanide to the feed, two techniques were applied: a 25-fold dilution of the influent and photocatalytic pre-treatment of the wastewater feed (Essam et al. 2014). In our work, the established algal–bacterial microcosm was efficient in removing up to 0.2 g L^−1^ KCN, 0.1 g L^− 1^ benzonitrile, and 0.5 g L^−1^ sodium salicylate from synthetic wastewater in a sustainable manner, without the need for the incorporation of additional biodegrading bacteria, pretreatment steps nor the supplementation of fertilizers such as NaHCO_3_ to the influent feed.

Biomonitoring is a test for the ability of a microorganism, weed, or plant to survive and/or grow in the presence of treated wastewater (Hybská et al. 2020). Algal growth in the presence of the toxic pollutants was a good indicator of the efficient treatment by the PBR. In algal toxicity testing, the growth of the isolated *Chlorella* strain was completely inhibited by 0.05 g L^− 1^ KCN, and the influent feed had 96% inhibition of its growth. However, in the continuous PBR, KCN concentration in the feed reached up to 0.2 g L^−1^ without harmful effects on algal growth, confirming the efficient detoxification of the pollutants by the system.

In fact, bioassays are essential to assess the effectiveness of bioremediation, as they evaluate the potential of environmental contaminants to interact with living organisms (Mazzeo et al. 2010). Unlike chemical analyses, which focus solely on measuring pollutant concentrations, bioassays provide insights into the bioavailability of substances in environmental samples. Phytotoxicity testing is usually used to assess the impact of various pollutants on seed germination and subsequent growth (Haq and Kalamdhad 2021). Particularly, *Lepidium sativum* is commonly used in phytotoxicity bioassays due to its sensitivity to contaminants such as heavy metals, petrochemicals, and polycyclic aromatic hydrocarbons (Janecka and Fijalkowski 2008). It is characterized by its rapid growth rate, adaptability to different humidity levels, and high contamination sensitivity. It has been previously applied to evaluate the phytotoxicity of composts (Aslam et al. 2008), sewage sludge (Mañas and De las Heras 2018), and soil saturated with wastewater (Mekki and Sayadi 2017), making it a versatile tool in environmental toxicity assessments. In the present study, the successful germination and stem elongation of the seeds, when incubated in the presence of effluent-treated water, confirmed the complete detoxification of the introduced pollutants in the algal–bacterial photobiorector.

The introduction of symbiotic bacteria to microalgae in biological treatment processses was shown to improve the algal flocculation properties, lipid content, and quality (Cho et al. 2015). The algal–bacterial flocs can be subsequently harvested and used in valuable biotechnological applications. The flocculation efficiency can be affected by the PBR operating conditions such as temperature, pH, and mixing speed (Ernest et al. 2017). Chitosan, as a flocculating agent, can also be used in water treatment since it is environmentally friendly, biodegradable, and readily accessible (Yang et al. 2016). In the present work, natural flocculation of the formed algal–bacterial flocks was satisfactory for the harvest of the effluent-treated wastewater. There was no need for the addition of flocculating agents or changing the operating conditions.

## Conclusions

Environmental pollution, particularly water contamination from industrial and agricultural activities, remains a significant global concern due to its detrimental impact on ecosystems and human health. This study successfully isolated a highly cyanide-degrading bacterial strain and a cyanide-tolerant algal strain from Egyptian municipal wastewater. The bacterial isolate demonstrated one of the highest cyanide degradation capacities reported in the past decade, showcasing its potential for industrial and environmental applications. To the best of our knowledge, this is the first study to integrate such a highly degrading *Bacillus licheniformis* MMT1 strain with an algal counterpart, a *Chlorella* strain, for the treatment of cyanide-containing compounds (potassium cyanide and benzonitrile) in a continuous photobioreactor (PBR) under simulated natural conditions. The constructed algal–bacterial PBR demonstrated remarkable efficacy, detoxifying synthetic wastewater containing the mixed cyanide compounds and sodium salicylate as a co-contaminant at significant concentrations within a short hydraulic retention time of 3.5 days under a 12/12-h light/dark cycle. These findings highlight the feasibility of employing photosynthetically aerated systems for the continuous treatment of industrial effluents in an eco-friendly and sustainable manner.

## Data Availability

The data for the 16S rRNA gene sequence analysis is available on the NCBI website. The sequences were deposited in the GenBank database with the accession number OM585600.
